# Probing recycled carbonate in the lower mantle

**DOI:** 10.1093/nsr/nwac061

**Published:** 2022-03-31

**Authors:** Li-Hui Chen, Xiao-Jun Wang, Sheng-Ao Liu

**Affiliations:** Department of Geology, State Key Laboratory of Continental Dynamics, Northwest University, China; Department of Geology, State Key Laboratory of Continental Dynamics, Northwest University, China; State Key Laboratory of Geological Processes and Mineral Resources, China University of Geosciences, China

## Abstract

Whether surficial carbonates can be carried into the Earth's lower mantle is key to global deep carbon cycles but remains poorly understood. New clues from magnesium and zinc isotopic systematics on rocks from deep-rooted mantle plumes are presented and discussed in this Perspective.

Carbonates in marine sediments and altered oceanic crust are major carbon reservoirs on Earth's surface, which can be transported into Earth's interior by subduction. High-pressure experiments and thermodynamic models demonstrate that carbonates can stably exist under Earth's lower mantle conditions [1,2]. However, melting experiments suggest that subducting carbonate-bearing oceanic crust will eventually undergo decarbonation melting at the mantle transition zone [3]. Therefore, whether there are some recycled carbonates in the lower mantle remains poorly understood. Fortunately, an answer has begun to take shape from the perspective of metal stable isotopes.

Given the considerable differences in δ^26^Mg (=[(^26^Mg/^24^Mg)_sample__/_(^26^Mg/^24^Mg)_DSM3_ − 1] × 1000) and δ^66^Zn (= [(^66 ^Zn/^64 ^Zn)_sample _/ (^66 ^Zn/^64 ^Zn)_JMC Lyon_ − 1] × 1000) between carbonates and the mantle [4], Mg and Zn isotopes of basalts can be used to trace recycled carbonates or carbonate-bearing materials in the mantle (see contributions in this Special Topic and Refs [4,5]). Many plumes stem from the core–mantle boundary and their derivant ocean island basalts (OIBs) can be regarded as a probe into the lower mantle. If carbonates can be transported into the lower mantle and captured by upwelling plumes, we will have an opportunity to find light Mg and heavy Zn isotopic anomalies in OIBs. The same is true for magmas from large igneous provinces (LIPs), which have also been regarded as plume products.

Pitcairn Island in the South Pacific Ocean and St. Helena Island in the South Atlantic Ocean are well known for the occurrence of EM1 (Enriched Mantle 1, characterized by unradiogenic Pb and Nd isotopic signatures) and HIMU (high μ, μ = ^238^U/^204 ^Pb, with extremely radiogenic Pb) OIBs, respectively. In general, recycled ancient pelagic sediments are thought to have contributed to the EM1 component in the lower mantle [6] while the recycling of ancient altered oceanic crust is critical for the formation of HIMU [7]. Given that both kinds of recycled crustal components may incorporate carbonates therein, lavas from these two islands are ideal candidates for probing potential recycled carbonates in the lower mantle.

Pitcairn OIBs have the lowest δ^26^Mg (as low as –0.4‰) so far in fresh OIBs [6] (Fig. 1a) that are distinct from the normal mantle. The low δ^26^Mg values are coupled with low Nb/Th and unradiogenic Pb and Nd isotopic ratios, which can be best explained if the Pitcairn EM1 source contains subducted carbonate-bearing pelagic sediments [6]. However, the low CaO/Al_2_O_3_ ratios of these EM1 basalts (Fig. 1b) contradict with experimental melts of carbonated mantle [8] and thus argue against any carbonates in the Pitcairn EM1 source. This paradox can be reconciled by early decarbonation reactions during subduction and the low-δ^26^Mg signature of carbonates has been inherited by silicate residue (e.g. eclogite), which ultimately became part of the Pitcairn plume [6]. Therefore, the low δ^26^Mg values of Pitcairn basalts do not support subduction of actual carbonate components into the lower mantle.

**Figure 1.**
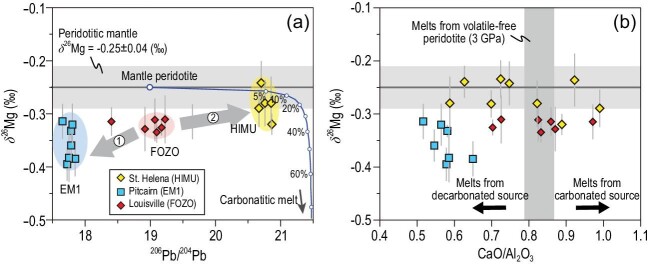
Variations in δ^26^Mg versus (a) ^206^Pb/^204^Pb and (b) CaO/Al_2_O_3_ ratio for different types of OIBs. In panel (a), the gray arrows denote general explanations for genesis of the two groups of OIBs: recycled (decarbonated) pelagic sediments (+ oceanic crust) for EM1 lavas (Arrow 1), carbonated peridotite source for HIMU lavas (Arrow 2). The blue curve shows the modeling for isotopic modification of a peridotitic mantle when it is metasomatized by carbonatitic melts with HIMU-type Pb isotopes. See Supplementary Data for data sources and modeling process.

Carbonated mantle has been regarded as the source of HIMU OIBs because (i) their low SiO_2_, high CaO and high CaO/Al_2_O_3_ (Fig. 1b) can be generated by melting of carbonated peridotite or eclogite [8], (ii) their trace element signatures are similar to those of carbonatitic melt inclusions in diamonds and (iii) olivine phenocrysts in HIMU OIBs have high Ca and low Al contents [7]. However, mantle-like δ^26^Mg values were observed in HIMU lavas (Fig. 1) [9] rather than frequently seen low-δ^26^Mg values in basalts generated by the melting of carbonated sources [4,5]. This is consistent with the metasomatism model for the generation of HIMU OIBs [7]: the HIMU mantle source could be carbonated peridotite formed by interaction between normal peridotite and carbonatitic melts from subducting carbonated oceanic crust. Such carbonatitic melts have MgO contents (typically <7.0 wt%; [3]) far lower than mantle peridotite, which cannot modify the latter's Mg isotopic compositions under a low melt/rock ratio condition (see the blue curve in Fig. 1a). Therefore, the St. Helena HIMU melts from carbonated peridotites still retain mantle-like δ^26^Mg values.

Recently, heavy δ^66^Zn values (0.31–0.38‰) have been found in Crozet OIBs and have been attributed to carbon-bearing oceanic crust in the sources [10]. However, it is unclear whether the major element (e.g. CaO and CaO/Al_2_O_3_) and trace element compositions (e.g. Hf/Sm ratio) of the Crozet OIBs are consistent with a carbonated source. Additionally, we still do not know whether the OIBs with a previously suggested carbonated source (e.g. HIMU OIB) have heavy δ^66^Zn. Therefore, it is too early to claim that the high δ^66^Zn values observed in several OIB samples are definite evidence for recycled carbonate in the deep mantle. For LIP-related rocks, Mg and Zn isotopic anomalies have not yet been observed in flood basalts. For example, picrites and basalts of the Emeishan LIP have mantle-like δ^26^Mg (–0.35‰ to –0.19‰) [11] and MORB-like δ^66^Zn (0.24–0.34‰) [12]. Considering their high-degree melting origin, the normal δ^26^Mg and δ^66^Zn values of flood basalts do not mean the lack of recycled carbonate in their sources because of enhanced dilution of melts from peridotitic mantle. Recently, low δ^26^Mg values (–1.09‰ to –0.35‰) have been observed from carbonatites and nephelinites (low-degree mantle melts) from the Tarim LIP in NW China [13], though their relationship to the mantle plume is not as clear as OIBs.

In summary, the ‘carbonate memory’, including low δ^26^Mg and high δ^66^Zn values, has been found in some OIBs, although there is still a certain distance to go in proving recycled carbonates in the lower mantle. Currently, published data of metal stable isotopes for fresh OIBs and LIP rocks are still limited. The fractionation behavior of these isotopes during melting has not been verified experimentally. There are still few integrated studies with measurements of multiple metal stable isotopes, radiogenic isotopes and major and trace elements on a same batch of OIB samples. These impedes the application of these metal stable isotopes to trace deep carbonate recycling. Nevertheless, with the further developments of analytical techniques, the expanding high-quality database of metal stable isotopes and the integrated geochemical study of plume-related lavas, we shall have a clear picture of deep carbonate recycling in the near future.

## Supplementary Material

nwac061_Supplemental_File
