# Supplementary material for: Probing recycled carbonate in the lower mantle
Source: Natl Sci Rev. 2022 Mar 31;9(6):nwac061. doi: 10.1093/nsr/nwac061 (PMC9166543; doi:10.1093/nsr/nwac061)
Supplement: nwac061_Supplemental_File [file nwac061_supplemental_file.docx]

**Supplementary materials for:**

**Probing recycled carbonate in the lower mantle**

*Li-Hui Chen (chenlh@nwu.edu.cn)*

Department of Geology, State Key Laboratory of Continental Dynamics, Northwest University, Xi’an 710069, China

*Xiao-Jun Wang (wangxj@nwu.edu.cn)*

Department of Geology, State Key Laboratory of Continental Dynamics, Northwest University, Xi’an 710069, China

*Sheng-Ao Liu (lsa@cugb.edu.cn)*

State Key Laboratory of Geological Processes and Mineral Resources, China University of Geosciences, Beijing 100083, China

**Data sources and quantitative modelling on Fig. 1:**

Pitcairn data are from Ref. [1]. Mg isotopic data for St. Helena basalts are from Ref. [2]. Major element and Pb isotopic data for St. Helena basalts are from Ref. [3] and Ref. [4], respectively. The Louisville basalts are commonly thought to derive from a normal peridotite source with FOZO (Focus Zone, [5])-type radiogenic isotope signatures [6, 7]. Major element and Mg isotope data for Louisville basalts are from Ref. [6], and Pb isotope data are from Ref [7]. Mg isotopic range for peridotitic mantle is from Ref. [8]. The CaO/Al_2_O_3_ range for experimental partial melts of volatile-free peridotite (3 GPa) is from Ref. [9].

On Fig. 1a, we conduct a quantitative calculation to evaluate the Mg isotopic variation of peridotitic mantle when it is metasomatized by carbonatitic melts (the blue curve). Here we assume the enriched endmember is a kind of carbonatitic melts with HIMU-type Pb isotopic compositions (^206^Pb/^204^Pb ratio = 21.5) and light Mg isotopic compositions (δ^26^Mg = −1.0 ‰). The Pb concentration (7 ppm) of such melts is after Ref. [10]. The MgO content (7.0 wt.%) of the carbonatitic melts is assumed to be equal to the highest MgO content of experimental carbonatitic melts from a carbonated eclogite [11]. We assume that the normal peridotitic mantle is a kind of FOZO-type mantle, and its ^206^Pb/^204^Pb ratio (^206^Pb/^204^Pb=19.0) is averaged from Louisville OIBs from Ref. [7]. Its Pb content (0.15 ppm) and MgO content (38.0 wt.%) are the same as the primitive mantle [12]. Mg isotopic composition (−0.25‰) of peridotitic mantle is from Ref. [8].

**References:**

1. Wang XJ, Chen LH, Hofmann AW, et al. Recycled ancient ghost carbonate in the Pitcairn mantle plume. Proc. Natl. Acad. Sci. U.S.A. 2018; 115: 8682-8687.
2. Wang XJ, Chen LH, Hanyu T, et al. Magnesium isotopic fractionation during basalt differentiation as recorded by evolved magmas. Earth. Planet. Sci. Lett. 2021; 565: 116954.
3. Kawabata H, Hanyu T, Chang Q, et al. The petrology and geochemistry of St. Helena alkali basalts: Evaluation of the oceanic crust-recycling model for HIMU OIB. J. Petrol. 2011; 52: 791-838.
4. Hanyu T, Kawabata H, Tatsumi Y, et al. Isotope evolution in the HIMU reservoir beneath St. Helena: Implications for the mantle recycling of U and Th. Geochim. Cosmochim. Acta 2014; 143: 232-252.
5. Hart SR, Hauri EH, Oschmann LA, et al. Mantle Plumes and Entrainment: Isotopic Evidence. Science 1992; 256: 517-520.
6. Zhong Y, Chen LH, Wang XJ, et al. Magnesium isotopic variation of oceanic island basalts generated by partial melting and crustal recycling. Earth. Planet. Sci. Lett. 2017; 463: 127-135.
7. Shi JH, Zeng G, Chen LH, et al. An eclogitic component in the Pitcairn mantle plume: evidence from olivine compositions and Fe isotopes of basalts. Geochim. Cosmochim. Acta 2022; 318: 415-427.
8. Teng FZ, Li WY, Ke S, et al. Magnesium isotopic composition of the Earth and chondrites. Geochim. Cosmochim. Acta 2010; 74: 4150-4166.
9. Walter MJ. Melting of Garnet Peridotite and the Origin of Komatiite and Depleted Lithosphere. J. Petrol. 1998; 39: 29-60.
10. Zeng, G, Chen LH, Hofmann AW, et al. Nephelinites in eastern China originating from the mantle transition zone. Chemical Geology. 2021; 576: 120276.
11. Thomson, A. R., Walter, M. J., Kohn, S. C. et al. Slab melting as a barrier to deep carbon subduction. Nature. 2016; 529: 76-79.
12. McDonough, W. F. and Sun, S. s. The composition of the Earth. Chemical Geology. 1995; 120: 223-253.
